# Music Familiarity Affects EEG Entrainment When Little Attention Is Paid

**DOI:** 10.3389/fnhum.2018.00444

**Published:** 2018-11-06

**Authors:** Yuiko Kumagai, Ryosuke Matsui, Toshihisa Tanaka

**Affiliations:** ^1^Department of Electrical and Electronic Engineering, Tokyo University of Agriculture and Technology, Tokyo, Japan; ^2^RIKEN Center for Brain Science, Saitama, Japan; ^3^RIKEN Center for Advanced Intelligence Project, Tokyo, Japan

**Keywords:** music, entrainment, familiarity, attention, electroencephalogram (EEG), spectrum analysis

## Abstract

To investigate the brain's response to music, many researchers have examined cortical entrainment in relation to periodic tunes, periodic beats, and music. Music familiarity is another factor that affects cortical entrainment, and electroencephalogram (EEG) studies have shown that stronger entrainment occurs while listening to unfamiliar music than while listening to familiar music. In the present study, we hypothesized that not only the level of familiarity but also the level of attention affects the level of entrainment. We simultaneously presented music and a silent movie to participants and we recorded an EEG while participants paid attention to either the music or the movie in order to investigate whether cortical entrainment is related to attention and music familiarity. The average cross-correlation function across channels, trials, and participants exhibited a pronounced positive peak at time lags around 130 ms and a negative peak at time lags around 260 ms. The statistical analysis of the two peaks revealed that the level of attention did not affect the level of entrainment, and, moreover, that in both the auditory-active and visual-active conditions, the entrainment level is stronger when listening to unfamiliar music than when listening to familiar music. This may indicate that the familiarity with music affects cortical activities when attention is not fully devoted to listening to music.

## 1. Introduction

According to many studies, music affects the human brain (Menon and Levitin, 2005; Schellenberg, 2005). For example, Schellenberg (2005) demonstrated that participants' performance during a cognition test was improved by listening to music. Menon and Levitin (2005) showed that human emotion was changed by music as well. Moreover, brain responses to music can potentially serve as tools for controlling a computer or a device, rehabilitation, music recommendation systems, and so on. For example, Treder et al. (2014) proposed a method for classifying the musical instrument to which individual participants paid the most attention while listening to polyphonic music. Ramirez et al. (2015) proposed a neurofeedback approach that determined participants' emotions based on their electroencephalogram (EEG) data in order to mitigate depression in elderly people. However, the process by which the brain recognizes music has not yet been explicated.

To comprehend auditory mechanisms, event-related potentials (ERPs), such as mismatch negativity (MMN) (Näätänen et al., 1978), have been measured in numerous contexts in the music and speech domains. Previous studies have used deviant speech sounds (Dehaene-Lambertz, 1997), rhythmic sequences (Lappe et al., 2013), and melodies (Virtala et al., 2014) to elicit the MMNs. Other studies have focused on an auditory response to periodic sound stimuli, called the auditory steady-state response (ASSR) (Lins and Picton, 1995). It has been reported that the ASSR is elicited by the amplitude-modulation of speech (Lamminmäki et al., 2014) and the periodic rhythm of music (Meltzer et al., 2015).

Recent research on speech perception has focused on cortical entrainment to the sound being listened to Ahissar et al. (2001), Luo and Poeppel (2007), Aiken and Picton (2008), Nourski et al. (2009), Ding and Simon (2013), Doelling et al. (2014), Ding and Simon (2014), and Zoefel and VanRullen (2016). Cortical entrainment to the envelope of speech was observed in electrophysiological recordings, such as magnetoencephalograms (MEGs) (Ahissar et al., 2001), EEGs (Aiken and Picton, 2008), and electrocorticograms (ECoGs) (Nourski et al., 2009). Also, several studies have reported a correlation between entrainment and speech intelligibility (Ahissar et al., 2001; Luo and Poeppel, 2007; Aiken and Picton, 2008; Ding and Simon, 2013; Doelling et al., 2014).

Studies of music perception have reported that periodic stimuli, such as the beat, meter, and rhythm, induce cortical entrainment (Fujioka et al., 2012; Nozaradan, 2014; Meltzer et al., 2015). Moreover, a recent MEG study showed cortical entrainment to music (Doelling and Poeppel, 2015). A functional magnetic resonance imaging (fMRI) study demonstrated that entrainment can occur as a result of the emotion and rhythm of music (Trost et al., 2014). Many studies have also investigated entrainment to emotions while listening to music in several types of contexts (see the review paper by Trost et al., 2017).

Another central factor of music perception is familiarity. Several brain imaging studies have investigated the brain regions activated while participants listen to familiar music, such as Satoh et al. (2006), Groussard et al. (2009), and Pereira et al. (2011). Some EEG studies have also demonstrated that a deviant tone included in a sequence of familiar tones yielded stronger MMN than that included in a sequence of unfamiliar sounds (Jacobsen et al., 2005). Moreover, a deviant chord in a sequence of familiar chords has been shown to elicit a greater response in the cross-correlation between the EEG and the music signals than that in a sequence of unfamiliar chords (Brattico et al., 2001). Meltzer et al. (2015) reported that the cerebral cortex induced a stronger response to the periodic rhythm of unfamiliar music than to that of familiar music. Furthermore, in a previous study, we reported a stronger response to unfamiliar music than to familiar music (Kumagai et al., 2017). Familiarity has also been studied extensively in research on visual perception. Interestingly, some studies have suggested that familiarity modulates attention. For example, it has been reported that selective attention to audiovisual speech cues is affected by familiarity (Barenholtz et al., 2016). Another study showed that during face recognition, unfamiliar faces resulted in a greater reduction of attention than did familiar faces (Jackson and Raymond, 2006).

Studies of auditory perception have investigated attention-dependent entrainment to speech or the beat. It has been observed that while listening to two speech samples at the same time, entrainment to the attended speech is stronger than that to the unattended one (Power et al., 2012; Horton et al., 2013). Moreover, it was demonstrated that the brain's response to the beat of music was stronger when listening to the beat compared to when reading sentences while ignoring the beat (Meltzer et al., 2015). Thus far, however, there has been little discussion concerning attention and music familiarity.

Following our previous work on familiarity-dependent entrainment and the aforementioned studies of attention-dependent entrainment, we hypothesized that entrainment is affected by attention with respect to the familiarity of music. Therefore, in this paper, we investigated how cortical entrainment is related to attention and music familiarity. To test this hypothesis, we recorded EEGs during three tasks: visual-active, auditory-active, and control. The participants were instructed to either listen to music or watch a silent movie. For analysis, cross-correlation functions between the envelope of the music and the EEG were calculated, and we compared cross-correlation values across the tasks and levels of familiarity.

## 2. Materials and methods

### 2.1. Participants

Fifteen males (mean age 23.1 ± 1.11; range 21–25 years old) who had no professional music education participated in this experiment. All participants were healthy; none reported any history of hearing impairment or neurological disorders. They signed written informed consent forms, and the study was approved by the Human Research Ethics Committee of the Tokyo University of Agriculture and Technology.

### 2.2. Experimental design

#### 2.2.1. Stimuli

We used musical stimuli (MIDI) synthesized by Sibelius (Avid Technology, USA), a music computation and notation program. We created forty-five pieces that consisted of melodies produced by piano sounds without harmony, as shown in the first and second columns of Table 1. An example of musical notes for the MIDI signal synthesized with the Sibelius software (Tchaikovsky, March from *the Nutcracker*) is presented in the Supplementary Material.

**Table 1:** Music for the audio stimuli.

**Composer**	**Title**	**Familiar**	**Unfamiliar**	**Disagreed**
L. v. Beethoven	Symphony No. 9 “Ode to Joy”	14	0	0
G. Bizet	Carmen “Toreador Song”	13	0	1
J. Brahms	Hungarian Dance No. 5	14	0	0
F. F. Chopin	Minute Waltz	10	3	1
A. Dvorak	Symphony No. 9 “New World”	14	0	0
S. E. W. Elgar	Pomp and Circumstance Marches	14	0	0
E. H. Grieg	In the Hall of the Mountain King	12	2	0
G. F. Handel	Messiah “Hallelujah”	13	1	0
G. Holst	Planets “Mercury”	13	1	0
F. Mendelssohn	Wedding March	13	0	1
W. A. Mozart	Eine Kleine Nachtmusik	13	1	0
W. A. Mozart	Piano Sonata No. 11–3 “Turkish March”	14	0	0
H. Necke	Csikos Post	14	0	0
J. Offenbach	Orpheus in the Underworld	13	1	0
J. Pachelbel	Canon	13	1	0
S. S. Prokofiev	Romeo and Juliet “Montagues and Capulets”	12	1	1
G. A. Rossini	William Tell Overture	14	0	0
E. A. L. Satie	Gymnopedie No. 1	2	12	0
E. A. L. Satie	Je te veux	6	8	0
J. Strauss	Voices of Spring Waltz	6	4	4
P. I. Tchaikovsky	Swan Lake “Scene”	14	0	0
P. I. Tchaikovsky	The Nutcracker “March”	14	0	0
P. I. Tchaikovsky	The Nutcracker “Waltz of the Flowers”	8	4	2
I. Albeniz	Piano Sonata Op. 82	0	14	0
L. v. Beethoven	Piano Sonata Op.14–1	0	14	0
A. Diabelli	Sonatina Op.151–2	2	12	0
A. Dvorak	Waltz	0	14	0
A. Dvorak	Serenade for Strings Op. 22–3 “Scherzo”	1	13	0
A. Dvorak	Serenade for Strings Op. 22–5 “Finale”	0	14	0
G. U. Faure	Dolly Suite Op. 56 “Kitty-valse”	2	11	1
E. H. Grieg	Lyric Pieces Op. 47–6 “Spring Dance”	0	13	1
F. J. Haydn	Piano Sonata No. 12	0	14	0
F. J. Haydn	Piano Sonata No. 28	0	14	0
F. J. Haydn	Piano Sonata No. 33	1	13	0
F. Kuhlau	Sonatina Op. 55–1	0	13	1
T. Leschetizky	Humoresque	0	14	0
F. Mendelssohn	Songs without Words Op. 19–1	0	14	0
W. A. Mozart	Piano Sonata KV309	1	12	1
S. S. Prokofiev	10 Pieces Op. 12–2 Gavotte	1	13	0
S. S. Prokofiev	10 Pieces Op. 12–3 Rigaudon	0	14	0
F. P. Schubert	Piano Sonata No. 4 Scherzo	0	14	0
F. P. Schubert	Piano Sonata No. 6–3	0	12	2
F. P. Schubert	String Quartet No. 4	0	14	0
F. P. Schubert	String Quartet No. 3	1	11	1
W. R. Wagner	Piano Sonata Op. 1	0	13	1

*Forty-five segments of the original version were extracted based on the music mentioned in this table. The last three columns present the frequencies of familiarity based on the questionnaire: familiar/unfamiliar/disagree. “Disagree” indicates the number of participants who provided different answers in the auditory-active and control tasks*.

The sound intensities of all of the generated musical pieces were identical. The length of each musical piece was 34 s with the tempo set to 150 beats per minute (bpm) (i.e., the frequency of a quarter of a note was 2.5 Hz). The sampling frequency was set to 44,100 Hz.

Sixty TV commercials from a DVD (“Masterpieces of CM in the World Volume 5,” Warner Music Japan) were selected. We presented the TV commercials with Japanese subtitles and without sound as visual stimuli.

#### 2.2.2. Procedure

**Figure 1:**
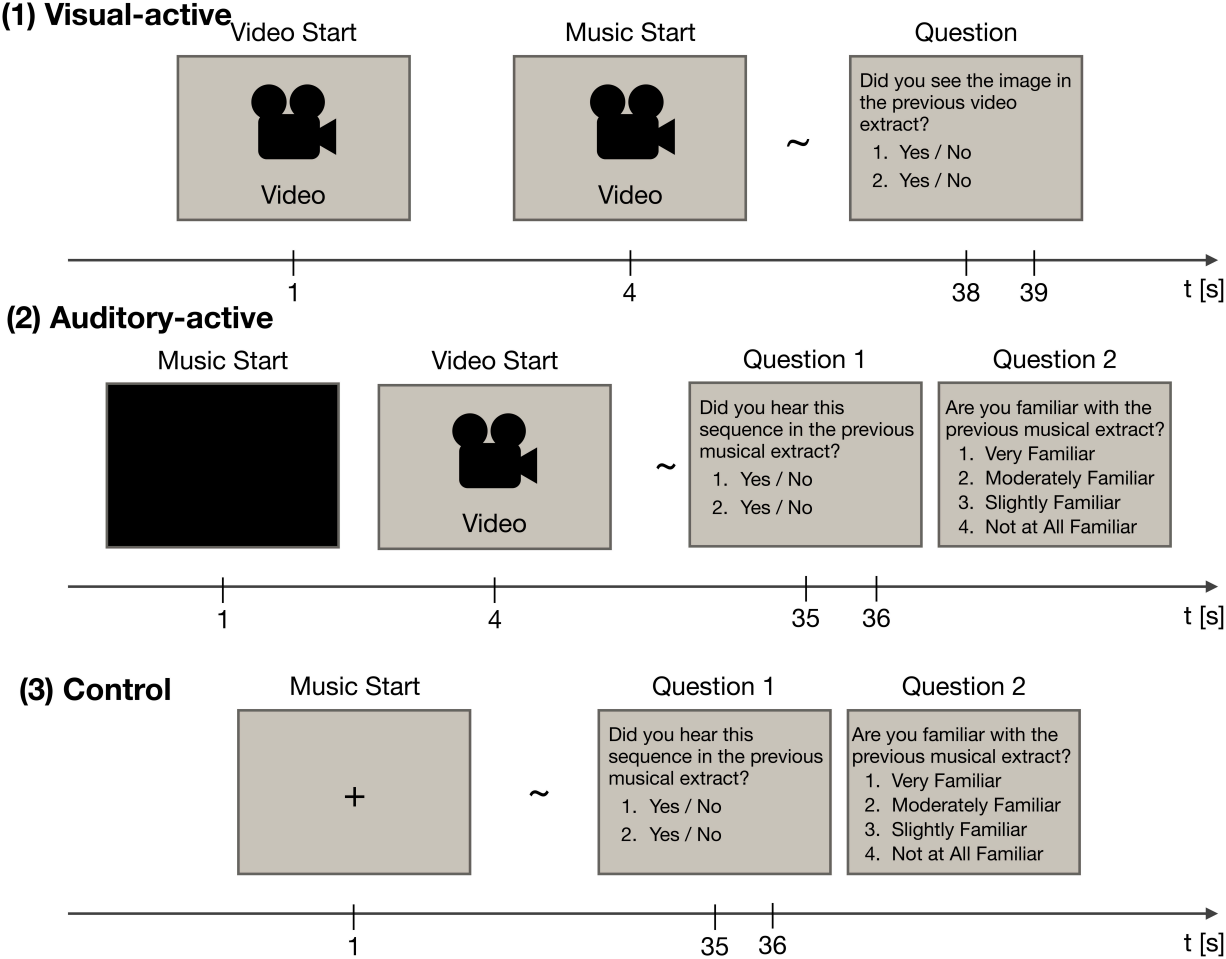
The experimental paradigm, consisting of three tasks: visual-active, auditory-active, and control. Each task was divided into thirty trials. In each trial, EEG recordings were acquired. Each of the thirty trials employed a different stimulus at random.

The experiment consisted of three tasks: visual-active, auditory-active, and control. The experimental paradigm is shown in Figure 1. Throughout the experiment, participants were instructed to watch an LCD monitor (ProLite T2735MSC, Iiyama, Japan). EEG recordings were made while they paid attention to either the visual or musical stimuli (i.e., watching the movie or listening to the music). Each task included thirty trials (for a total of ninety trials). Forty-five musical stimuli were presented in two of the three tasks, and video stimuli were presented in two of the three tasks. The order of the stimuli was random across the tasks.

At the end of each trial, the participants were asked two questions about the stimuli to confirm whether they paid attention to the stimuli. In the auditory-active and control tasks, they were also asked whether they were familiar with the presented music. In this question, two short segments of the musical stimuli (3 s) or two frames of the video stimuli were presented. Participants also answered two yes/no questions; the questions asked whether the choices had been included in the stimuli of the trial. Incorrect choices were made by selecting the music passage that had not been used as the musical stimulus. The rate of “yes” answers in each task was 50%. Although we used the musical stimuli twice in total, the choices were different across the tasks. In the auditory-active and control tasks, the participants were instructed to rate their level of familiarity with the musical stimuli on a 4-point scale (i.e., “very familiar,” “moderately familiar,” “slightly familiar,” and “not at all familiar”). Details of the three tasks are given below.

##### 2.2.2.1. The visual-active task

In the visual-active task, a 37-s-long silent video with subtitles was presented 1 s after the onset of the trial. Then, at 4 s, one of the musical stimuli began. Participants were instructed to focus on the video stimulus and to ignore the musical stimulus. After the end of the video stimulus, two frames of the video stimulus were presented. Participants were asked whether the frames had been included in the presented video.

##### 2.2.2.2. The auditory-active task

In the auditory-active task, a 34-s-long musical stimulus was presented 1 s after the onset of the trial. Then, at 4 s, a silent video with subtitles began. Participants were instructed to focus on the musical stimulus and to ignore the video stimulus. After the end of the musical stimulus, two musical passages were presented, one that was included in the stimulus and one that was not included in the stimulus. Participants were asked whether the passages had been included in the presented music; the participants were also asked about their level of familiarity with the music.

##### 2.2.2.3. The control task

In the control task, a 36-s-long musical stimulus was presented 1 s after the onset of the trial. Participants were instructed to focus on the musical stimulus while fixating on the screen. After the end of the musical stimulus, two musical passages were presented, one that was included in the stimulus and one that was not included in the stimulus. Again, participants were asked whether the passages had been included in the presented music; the participants were also asked about their level of familiarity with the music.

### 2.3. Data acquisition

EEGs were measured using an EEG gel head cap with 64 scalp electrodes (Twente Medical Systems International [TMSi], Oldenzaal, The Netherlands) following the international 10–10 placement system (Fp1, Fpz, Fp2, F7, F3, Fz, F4, F8, FC5, FC1, FC2, FC6, T7, C3, Cz, C4, T8, CP5, CP1, CP2, CP6, P7, P3, Pz, P4, P8, POz, O1, Oz, O2, AF7, AF3, AF4, AF8, F5, F1, F2, F6, FC3, FCz, FC4, C5, C1, C2, C6, CP3, CPz, CP4, P5, P1, P2, P6, PO5, PO3, PO4, PO6, FT7, FT8, TP7, TP8, PO7, PO8, M1, and M2). For patient grounding, a wetted TMSi wristband was used. To measure eye movement, an electrooculogram (EOG) was recorded with two bipolar electrodes at the corner of the right eye (referenced to the right ear) and placed above the right eye (referenced to the left ear). All channels were amplified using a Refa 72-channel amplifier (TMSi) against the average of all connected inputs. The signals were sampled at a sampling rate of 2,048 Hz, and they were recorded with TMSi Polybench. At the same time, we recorded the audio signals to validate the onset timing of the presented musical stimuli.

### 2.4. Data analysis

#### 2.4.1. Labeling

After the experiment, the musical stimuli were categorized for each participant, according to the answers of the participants in the auditory-active and control tasks. Trials in which participants answered “very familiar” and “moderately familiar” were labeled as “familiar,” and trials in which participants answered “slightly familiar” and “not at all familiar” were labeled as “unfamiliar.”

#### 2.4.2. Preprocessing

We analyzed the relationship between the envelope of the musical stimuli and the EEG. One participant (s5ka) was excluded from the analysis because, as a result of technical difficulties, the audio signals related to this participant could not be recorded. First, a zero-phase second-order infinite impulse response notch digital filter (50 Hz) and a zero-phase fifth-order Butterworth digital highpass filter (1 Hz) were applied to the recorded EEG. Second, the trials contaminated with a large amount of artifacts were removed by visual inspection. Third, to remove artifacts caused by EOG, we applied a blind source separation algorithm called second-order blind identification to the recorded EEGs (Belouchrani et al., 1993; Belouchrani and Cichocki, 2000; Cichocki and Amari, 2002). We then re-referenced the filtered EEGs from the average reference to the average of ear references (M1 and M2). Moreover, the EEGs filtered by a low-pass filter with a cutoff frequency of 100 Hz were downsampled to 256 Hz. Finally, a zero-phase fifth-order Butterworth digital bandpass filter between 1 and 40 Hz was applied.

For the musical stimuli, the original music signals were first resampled from 44,100 to 8,192 Hz. Thereafter, the envelopes of the resampled musical stimuli were calculated using the Hilbert transform; the envelopes were then filtered by a low-pass filter with a cutoff frequency of 100 Hz and were downsampled to 256 Hz. Finally, a zero-phase fifth-order Butterworth digital bandpass filter between 1 and 40 Hz was applied to the envelope.

#### 2.4.3. Supervised classification of EEGs in attentional conditions

To show the electrophysiolgical differences of the responses to the visual-active and auditory-active tasks, we employed a support vector machine (SVM) with a common spatial pattern (CSP) (Ramoser et al., 2000). This approach is used extensively for a two-class classification problem in brain-computer interfaces (BCIs). The pre-processed EEGs (29 s) were divided into 4-s epochs. Each epoch was labeled either as visual-active or auditory-active. The data were divided into a training dataset and a test dataset, and each epoch was projected to a vector consisting of six log-variance features by CSPs corresponding to the first three largest eigenvalues and the last three smallest eigenvalues. The classification accuracy of the SVM was calculated based on a five-fold cross-validation. The SVM was implemented with scikit-learn from Python with a Gaussian kernel function.

The next step was to randomly shuffle the data of all epochs and divide them into two datasets, where the 50% of epochs was labeled as visual-active and the rest was labeled as auditory-active. In the same manner, the classification accuracy was calculated based on a five-fold cross-validation. This trial was independently run 5,000 times.

#### 2.4.4. Cross-correlation functions

The cross-correlation function is widely used for evaluating the spectro-temporal characteristics of the entrainment between the cortical response and the stimulus (Lalor et al., 2009; VanRullen and Macdonald, 2012). To calculate the cross-correlation function, 29-second epochs of the filtered EEGs and music (from 1 s after the end of the trial to avoid edge effects from the filter) were used. The cross-correlation functions between the envelope of the sound stimuli, Envelope(*t*), and the *n*th EEG channel, EEG_*n*_(*t*), from *t* = *T*_1_ to *t* = *T*_2_ are given as follows:

(1) $$\begin{array}{r} r_{n}\left(\tau \right)=\overset{T_{2}}{\underset{t=T_{1}}{\sum }}\text{Envelope}\left(t\right)\text{EE}{\text{G}}_{n}\left(t+\tau \right), \end{array}$$

where τ denotes the time lag between the envelope and the EEG signal, and both signals are normalized to the zero mean and unit variance. The time lags to be analyzed were set between *T*_1_ = −0.6 and *T*_2_ = 0.6 s, defining the cross-correlation values for a little over a second to include the minimum frequency of 1 Hz of the analyzing band-passed signals. The negative parts of the lags were used to confirm whether or not the pronounced peaks were commonly higher than the baseline (Kumagai et al., 2017). Scalp topographic maps of the cross-correlation values at the peaks were drawn with the open-source MATLAB toolbox EEGLAB (Delorme and Makeig, 2004).

#### 2.4.5. Evaluation

As suggested by Zoefel and VanRullen (2016) and Kumagai et al. (2017), we evaluated the pronounced peak values appearing in the cross-correlation functions for each task (i.e., visual-active, auditory-active, or control) as well as the level of familiarity (familiar or unfamiliar). To determine the peak time lag, the cross-correlation functions were first averaged across all participants, channels, and trials. Then, the maximum value of the grand average of the cross-correlation functions was detected as the positive peak value, and the minimum value of the functions was detected as the negative peak value. For the peak values, we conducted two types of evaluation tests. First, in order to examine whether the peak values differed from zero (reflecting significant entrainment to the musical stimuli), we compared our cross-correlation results accordingly with surrogate distributions; the surrogate distributions were given as cross-correlation functions between an EEG drawn from the trials and the envelope drawn from the trials–except the one used for the EEG (7,500 times). We calculated *p*-values for each time lag using the cross-correlation values averaged across the channels of surrogate distributions. We calculated “real” cross-correlation functions under the null hypothesis that the average channel value of real distributions is equal to that of surrogate distributions. Sample sizes of real distributions were the number of trials (i.e., about 170 trials by each task type and level of familiarity). Second, to examine the peak value across the tasks (i.e., visual-active, auditory-active, and control) and level of familiarity (i.e., familiar and unfamiliar), a two-way repeated-measures analysis of variance (ANOVA) was performed. The task and level of familiarity were defined as the independent variables, and the peak value was introduced as the dependent variable. The ANOVA test was performed using the averaged data points across the channels for an individual participant. Therefore, the sample size for the ANOVA was the same as the number of participants. When the assumption of sphericity was violated, we corrected the degrees of freedom using the Greenhouse-Geisser correction. The effect size was calculated as generalized eta squared $\left(\eta_{G}^{2}\right)$ (Olejnik and Algina, 2003; Bakeman, 2005).

## 3. Results

### 3.1. Behavioral results

**Figure 2:**
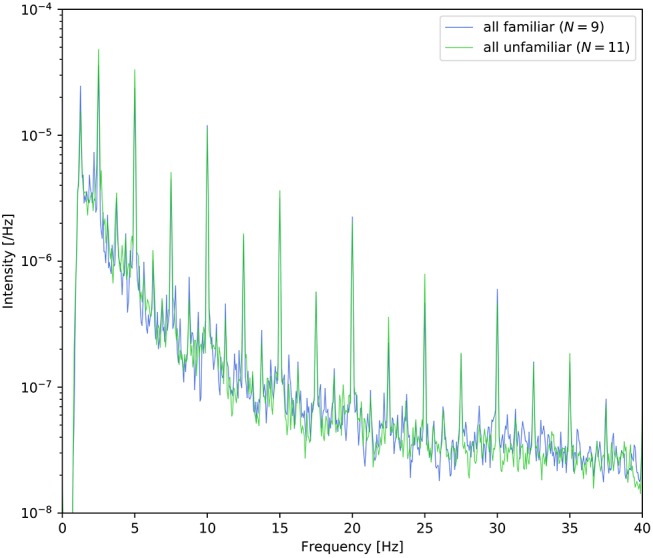
Power density spectra of the envelope averaged across the nine tunes for which all participants answered “familiar” and the eleven tunes for which all participants answered “unfamiliar.”

At the end of each trial, the participants were asked to answer two yes/no comprehension questions for the presented video or musical piece. The average comprehension accuracy is summarized in Table 2. All participants answered these questions satisfactorily, and these results suggest that the participants successfully attended to the target stimuli as instructed. In the three right columns (familiar/unfamiliar/disagree) of Table 1, the frequencies of familiarity are shown based on the questionnaire. “Disagree” indicates the number of participants who gave different answers in the auditory-active and control tasks. The detailed counts of familiarity level for each task are listed in the Supplementary Material. Moreover, as shown in Figure 2, it appears that there was no difference between the spectra of familair and unfamiliar tunes where the spectrum of familiar music is the averaged amplitude of music for which all the participants gave the same answer.

**Table 2:** Accuracy of the answers.

**Task**	**Accuracy [%]**
Visual-active	97.3 ± 2.78
Audiroty active	87.7 ± 6.14
Control	86.4 ± 4.14

### 3.2. Classification results

**Figure 3:**
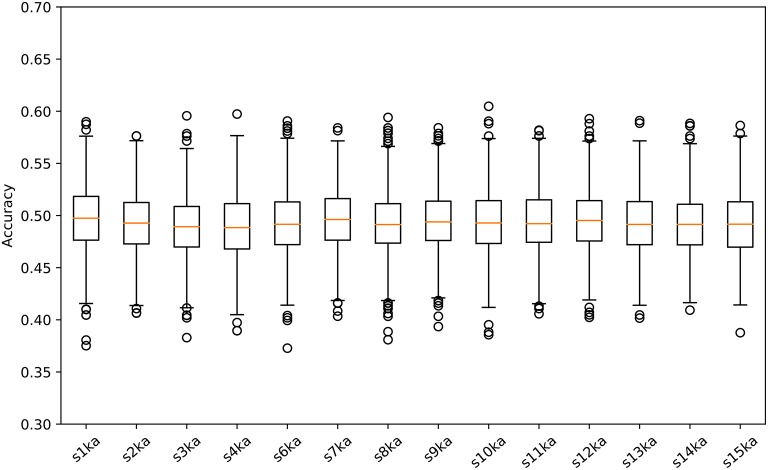
Box plots of the classification accuracies for the randomly shuffled datasets.

We conducted a two-class classification of the EEG (with labels of the visual-active and the auditory-active tasks) by the SVM using log-variance features of the CSP. Table 3 shows the results of the classification accuracy for each participant. Twelve of fourteen participants archieved an accuracy of 100%, and the remaining two participants reached almost 100%. This is not surprizing results. Distribution of the log-variance features projected by CSPs derived from the data for each participant is illustrated in Supplementary Materials, where features of two tasks are clearly separated. To validate these results, we conducted the same way of the classification for the randomly shuffled datasets. Figure 3 illustrates box plots of the classification accuracies. For all subjects, the accuracies were distributed around the chance level (50%). This implies that it is very probable that the level of attention is the difference between the visual-active and auditory-active tasks.

**Table 3:** Accuracy of the two-class classification by SVM with the CSP log-variance feature.

**s1ka**	**s2ka**	**s3ka**	**s4ka**	**s6ka**
1.000 ± 0.000	1.000 ± 0.000	1.000 ± 0.000	0.967 ± 0.024	1.000 ± 0.000
s7ka	s8ka	s9ka	s10ka	s11ka
1.000 ± 0.000	1.000 ± 0.000	0.997 ± 0.006	1.000 ± 0.000	1.000 ± 0.000
s12ka	s13ka	s14ka	s15ka	
1.000 ± 0.000	1.000 ± 0.000	1.000 ± 0.000	1.000 ± 0.000	

*The result is the average and the standard error of five-fold cross-validation*.

### 3.3. Cross-correlation functions

**Figure 4:**
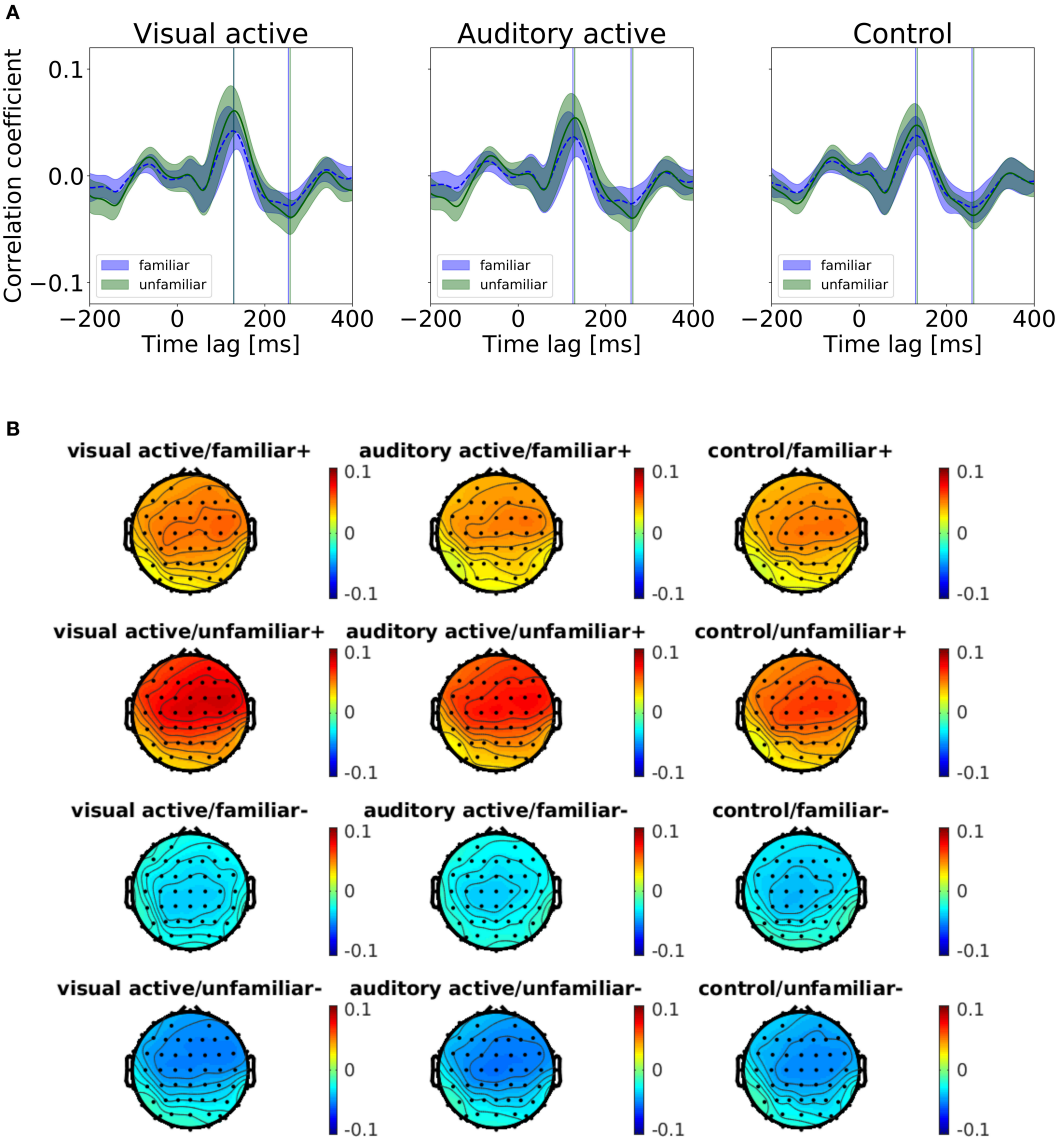
**(A)** Results of the cross-correlation values averaged across the channels. Cross-correlation values between the envelopes of the sound stimuli and the EEGs averaged across the trials and participants for the task and the level of familiarity. The solid line indicates the grand average of the cross-correlation values across channels. The shaded region indicates the standard error. The vertical lines indicate the maximum (i.e., positive peaks) and the minimum (i.e., negative peaks) of the cross-correlation values of the averaged cross-correlation functions. **(B)** Each subfigure shows the peaks at the time lags around 130 and 260 ms. The topographies show the distribution of the cross-correlation values at the positive (+) and negative (−) peaks.

We categorized the trials of the auditory-active and control tasks as familiar or unfamiliar according to the participants' answers. Figure 4 shows the cross-correlation curves between the envelope of the musical stimuli and the EEGs for all channels averaged across the participants, the trials, and the standard deviations. The solid line represents the grand average of the cross-correlation values across the channels and participants for each task and level of familiarity, and the shaded region indicates the standard error across the participants. The cross-correlations of the individual participants at electrodes Fz, FCz, and Cz are presented in the Supplementary Material. The two vertical lines indicate time lags that have the maximum and minimum values of the grand averaged cross-correlation functions (i.e., peaks). The time lags of the peaks are shown in Table 4. All plots show positive peaks at the time lags around 130 ms and negative peaks at the time lags around 260 ms. Moreover, in Figure 4, the topographies illustrate the distribution of the cross-correlation values at the positive and negative peaks for each task and level of familiarity. As Figure 4A illustrates, both peak values of the unfamiliar category were larger than those of the familiar category. In addition, it appears that there were no differences among the tasks. The topographical plots are presented in Figure 4. Although only the control task lacked visual stimuli, the topographical plots of the three tasks have similar distributions.

**Table 4:** Summary of time lags at the peaks (positive/negative).

**Time lag [ms]**		**Task**	
**Familiarity**	**Visual-active**	**auditory-active**	**Control**
Familiar	128.9/253.9	125.0/257.8	128.9/257.8
Unfamiliar	128.9/257.8	132.8/261.7	128.9/261.7

### 3.4. Statistical verifications

**Figure 5:**
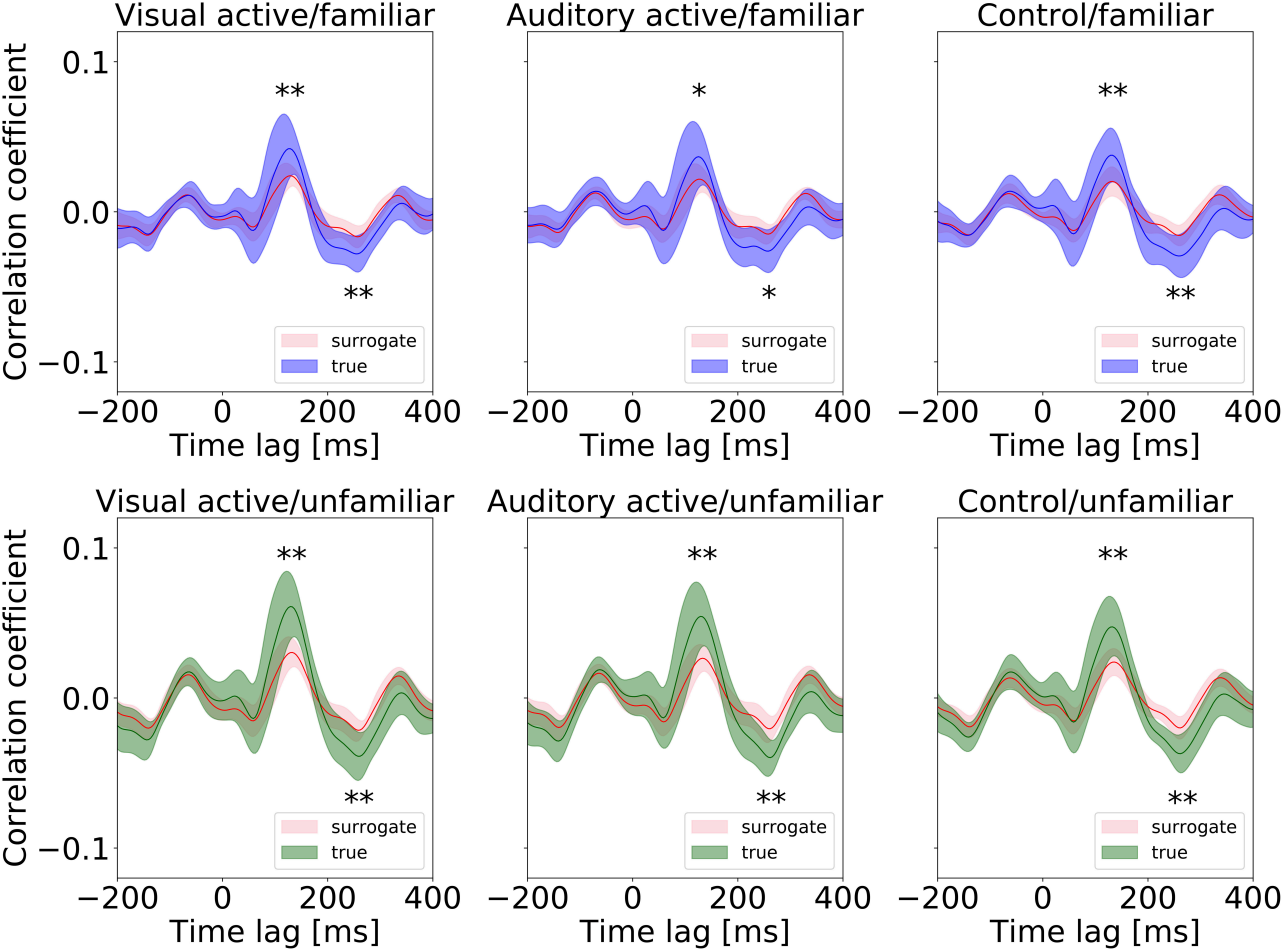
Surrogate distributions for all conditions. The cross-correlation functions shown in Figure 4 are also overlapped in each plot, and all the positive and negative peaks are significant in size (^*^ and ^**^ indicate *p* < 0.05 and *p* < 0.01, respectively).

First, we conducted a *t*-test to compare the cross-correlation results with the surrogate distributions at the peaks, as shown in Figure 5. At the positive and negative peaks in all conditions except “auditory-active/familiar,” *p* < 0.01 compared to the real cross-correlation values. This supports the existence of neural entrainment to music.

Second, we examined the cross-correlation changes across the tasks and levels of familiarity. We also performed two-way repeated-measures ANOVA tests (i.e., 3 tasks × 2-class familiarity) on the cross-correlation values averaged across all trials and channels at the two peaks. A summary of the results is shown in Table 5. The repeated-measures ANOVA test for the positive peak (around 130 ms) yielded a significant main effect due to the level of familiarity, *F*_(1, 13)_ = 67.3840, *p* = 0.0000, $\eta_{G}^{2}=0.1271$. In contrast, there was no significant main effect due to the task, *F*_(1.46, 19.01)_ = 3.3941, *p* = 0.0675. There was also no significant interaction between the task and the level of familiarity, *F*_(1.64, 21.27)_ = 3.2299, *p* = 0.0681. For the negative peak, the same test was performed, and the same results were obtained, that is, the repeated-measures ANOVA test on the negative peak (around 260 ms) showed a significant main effect due to the level of familiarity, *F*_(1, 13)_ = 30.7389, *p* = 0.0001, $\eta_{G}^{2}=0.1335$. In contrast, there was no significant main effect due to the task, *F*_(1.66, 21.57)_ = 0.0136, *p* = 0.9744. There was also no significant interaction between the task and the level of familiarity, *F*_(1.78, 23.15)_ = 1.3241, *p* = 0.2828. In summary, the results revealed that the cortical responses to unfamiliar music were significantly stronger than those to familiar music, and no significant difference was confirmed between the cortical responses in the different tasks.

**Table 5:** Summary of the ANOVA tests.

**Peak**	**Effect of task**	**Effect of familiarity level**	**Effect of interaction**
Positive peak	*p* = 0.0675	*p* = 0.0000^**^	*p* = 0.0681
Negative peak	*p* = 0.9744	*p* = 0.0001^**^	*p* = 0.2828

*There were significant main effects due to the level of familiarity in all tasks at the averaged peaks, but there was no significant main effect due to the task or interaction*.

** *p < 0.01*.

## 4. Discussion

Significant main effects on the positive peaks due to the level of familiarity were observed through the grand average of the cross-correlation values. It was confirmed that compared to the unfamiliar music, the familiar music's grand average of the cross-correlation values was significantly smaller. This accords with our earlier observations (Kumagai et al., 2017), which showed that the response to unfamiliar music is stronger than that to familiar music. Our results also support the findings of Meltzer et al. (2015), who observed a stronger entrainment to the periodic rhythm of scrambled (nonsensical) music compared to familiar music. Moreover, it was confirmed that the level of attention did not significantly affect the cross-correlation values. In general, therefore, entrainment to unfamiliar music occurs more strongly than that to familiar music, regardless of the level of attention.

Figure 4 illustrates peaks in negative as well as positive time lags, and peaks in the unfamilar cases appear to be larger than peaks in the familar cases. It can be conceptualized that these peaks are due to the periodic structure of the music because the cross-correlation is the inverse Fourier transform of the product of two spectra. Therefore, the peaks from the negative time lags can be related to the steady-state responses; these responses are enhanced by unfamiliar music, as also reported by Meltzer et al. (2015).

This study's most obvious finding is that even in the visual-active task, the cross-correlation values at the two peaks were significantly larger when listening to the unfamiliar music than those when listening to the familiar music. This is despite the fact that in the visual-active task, the participants were instructed to watch a silent movie, which may have led the participant to pay less attention to the musical stimuli than to the visual stimuli. This indicates the possibility that unfamiliar information might enter the brain more easily than familiar information in an unthinking or unconscious manner.

The results of this study show that the positive peak of the cross-correlation functions in all tasks occurred at the time lags around 130 ms; the results also show that the negative peak occurred at the time lags around 260 ms. Meltzer et al. (2015), in a study in which the participants listened to the periodic rhythm of music, reported that the time delay was about 94 ms. Kong et al. (2014)'s investigation of entrainment to speech showed that the positive peak of the cross-correlation function between the EEG and speech occurred at the time lags around 150 ms and that the negative peak occurred at the time lags around 310 ms. Our positive peak times occurred later than those associated with listening to periodic rhythms; however, both peak times occurred earlier than those associated with listening to speech. To summarize the previous findings in research and our findings, entrainment to different auditory modalities may occur with different time lags. The response to periodic rhythm is faster than the response to music, which is faster than the response to speech. This difference in the time lags may be due to the complexity of auditory signals and the responding brain regions. A future study with brain imaging, such as fMRI, could verify the latter hypothesis.

The most interesting results of this study are the differences in entrainment at various attention levels. Several reports have shown that attention can modulate brain responses when participants pay attention to different sensory modalities. For instance, Meltzer et al. (2015) measured entrainment to the beat of music while attending to a musical stimulus and ignoring the auditory signal instead of reading a text. The study found that there was a significant increase in the amplitude of the beat frequency for the auditory-active condition compared to the visual-active condition. Contrary to the hypothesis, our results show no significant differences among the visual-active, auditory-active, and control tasks. Our results show that there were no differences between the auditory-active and control tasks; thus, the cross-correlation functions were not affected by the visual stimulus (i.e., a movie). In light of these observations, it can be concluded that when listening to music, familiarity enhances entrainment even when participants do not pay full attention to the music.

This paper investigated the relationship between familiarity and attention in relation to entrainment while participants listened to music consisting of melodies produced by piano sounds. Our hypothesis was that entrainment to familiar or unfamiliar music would be affected by attention. To test this hypothesis, we conducted an experiment in which participants were instructed to either listen to music or watch a silent movie. EEGs were recorded during the tasks, and we computed the cross-correlation values between the EEGs and the envelopes of the musical stimuli. The grand averages of the cross-correlation values at the positive and negative peaks when listening to the unfamiliar music were significantly larger than those when listening to the familiar music during all tasks. Thus, our hypothesis was rejected. This finding suggests that even when humans do not pay full attention to the presented music, the cortical response to music can be stronger for unfamiliar music than for familiar music.

Although the machine learning-based analysis showed the clear difference in the EEG data between the visual-active and auditory-active tasks, a limitation of this study might be the lack of behavioral measurements of the level of attention and a relatively low visual load. An improved design of visual stimuli and musical stimuli should be considered for a high visual load that can significantly reduce sensory processing of auditory stimuli (Molloy et al., 2015). It is necessary to understand the physiology under two types of attention levels (the visual-active and auditory-active tasks). This may need higher spatial or temporal resolution measurement such as fMRI (Johnson and Zatorre, 2005) and ECoG (Gomez-Ramirez et al., 2011).

## Author contributions

YK designed the experiment, collected data, contributed to analysis and interpretation of data, and wrote the initial draft of the manuscript. RM have contributed to data analysis and interpretation. TT designed the experiment, contributed to analysis and interpretation of data, and revised the draft of the manuscript. The final version of the manuscript was approved by all authors.

### Conflict of interest statement

The authors declare that the research was conducted in the absence of any commercial or financial relationships that could be construed as a potential conflict of interest.
