# Supplementary material for: Music Familiarity Affects EEG Entrainment When Little Attention Is Paid
Source: Front Hum Neurosci. 2018 Nov 6;12:444. doi: 10.3389/fnhum.2018.00444 (PMC6232314; doi:10.3389/fnhum.2018.00444)
Supplement: Supplementary file 1 [file Data_Sheet_1.PDF]

Supplementary Material:
Music Familiarity Affects EEG Entrainment When Little Attention Is Paid

Table S1. The number of samples of familiar and unfamiliar tunes. For some participants, the sum of familiar and unfamilar tunes is not 30. This is due to either the disagreement of the answers given in two differnt tasks (control and either auditory or visual-active) or the rejection of the trial caused by a low-quality EEG.

Task	Visual-active		Auditory-active		Control	
Participant	familiar	unfamiliar	familiar	unfamiliar	familiar	unfamiliar
s1ka	14	13	12	11	14	12
s2ka	14	14	16	14	14	13
s3ka	16	14	15	11	16	11
s4ka	14	16	11	13	12	17
s6ka	13	16	13	16	14	13
s7ka	12	17	14	12	13	11
s8ka	10	18	10	16	10	17
s9ka	12	16	13	16	13	17
s10ka	13	17	14	16	13	17
s11ka	13	17	12	13	10	16
s12ka	14	16	14	16	14	16
s13ka	12	17	15	15	12	16
s14ka	13	16	12	17	11	18
s15ka	14	15	13	14	10	17

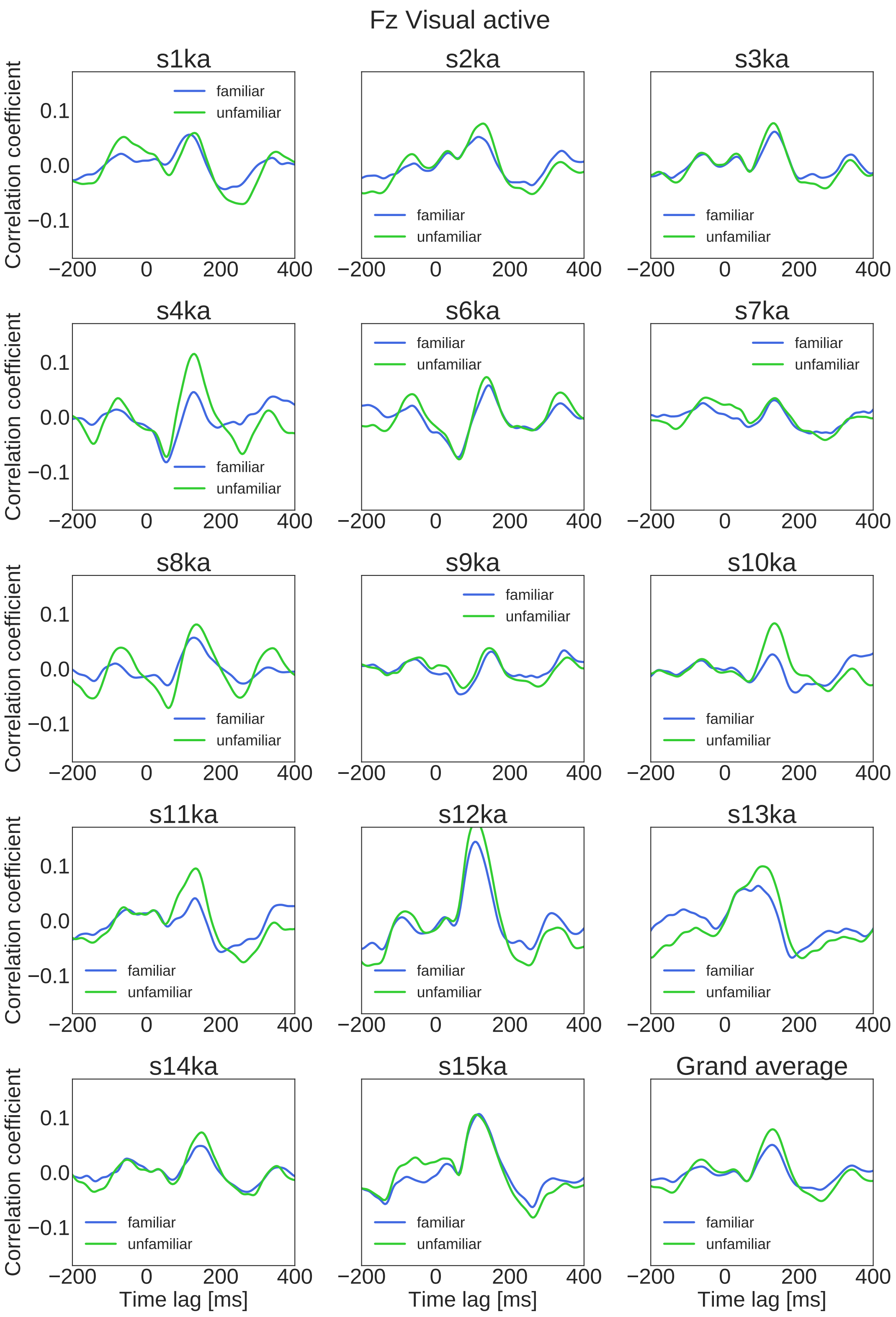

Figure S1. The averaged cross-correlation at Fz across trials in the visual-active condition for each participant and the grand average across participants.

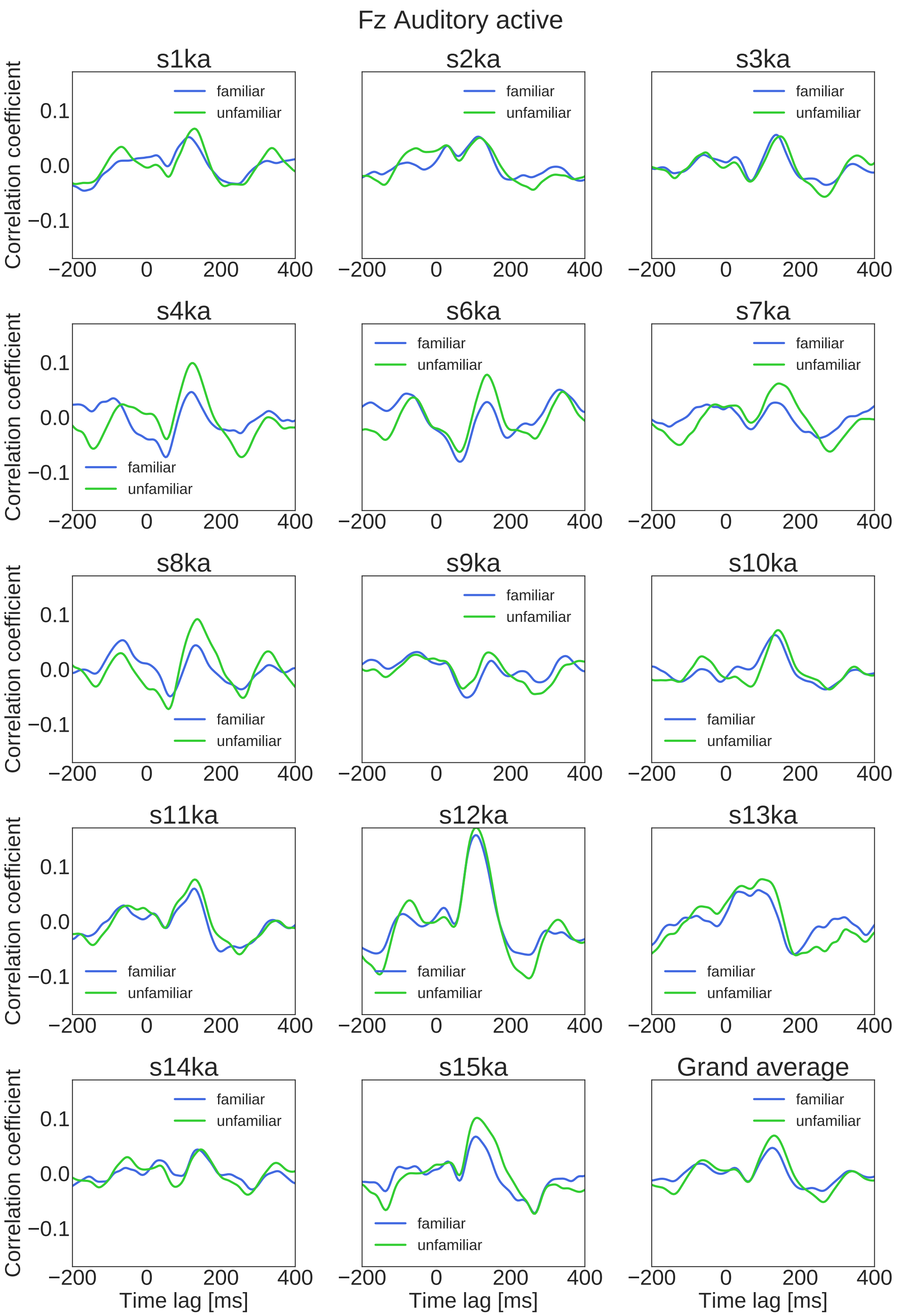

Figure S2. The averaged cross-correlation at Fz across trials in the auditory-active condition for each participant and the grand average across participants.

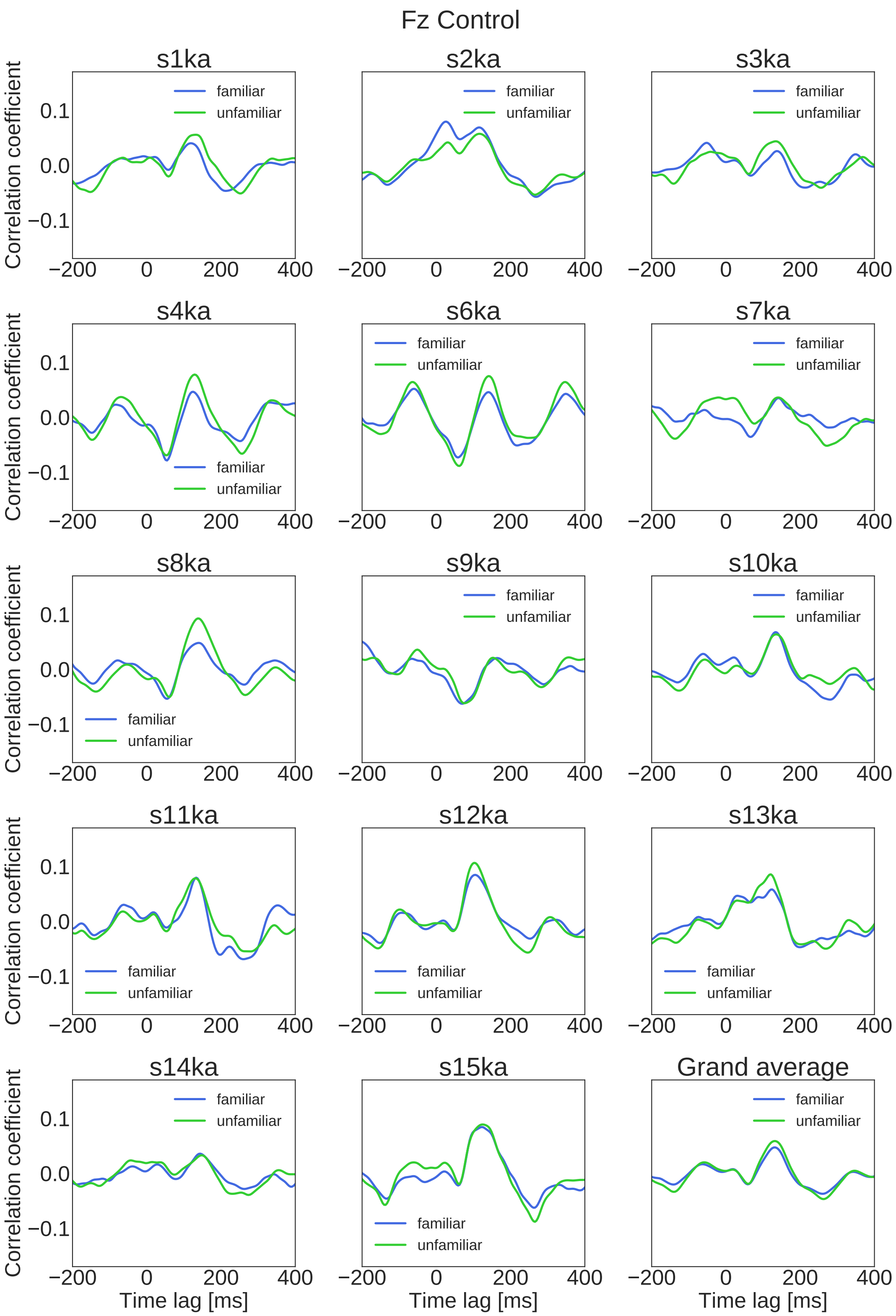

Figure S3. The averaged cross-correlation at Fz across trials in the control condition for each participant and the grand average across participants.

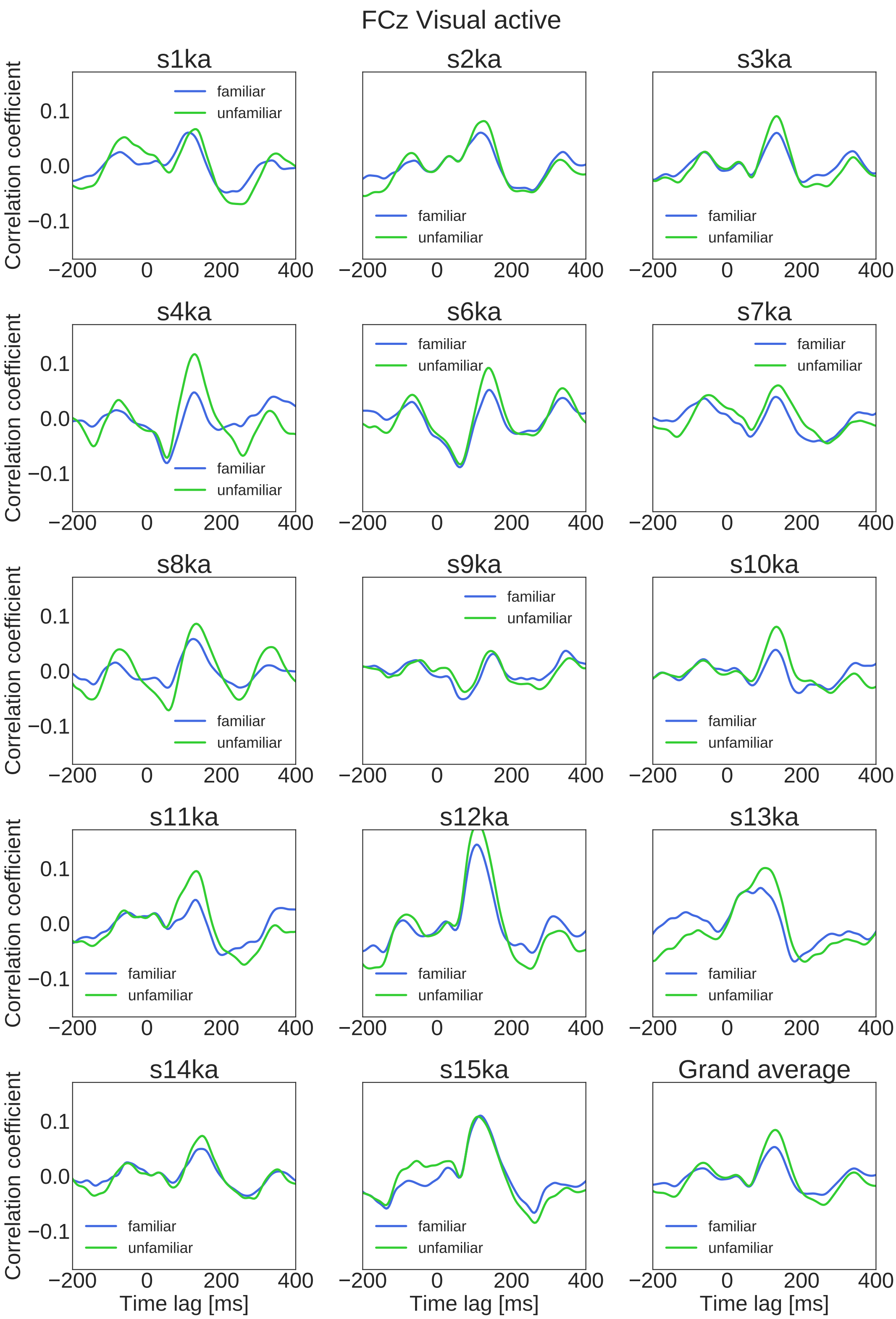

Figure S4. The averaged cross-correlation at FCz across trials in the visual-active condition for each participant and the grand average across participants.

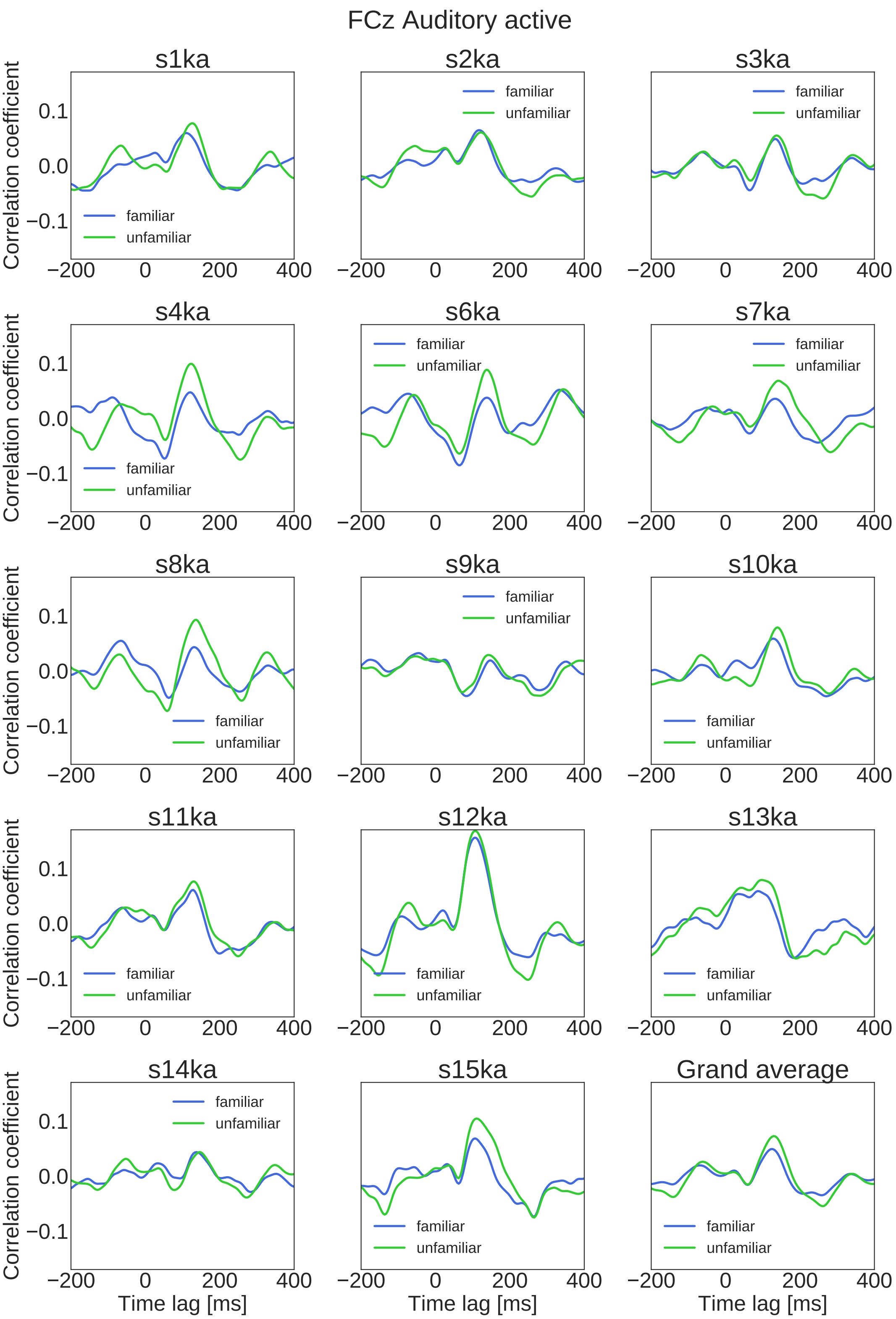

Figure S5. The averaged cross-correlation at FCz across trials in the auditory-active condition for each participant and the grand average across participants.

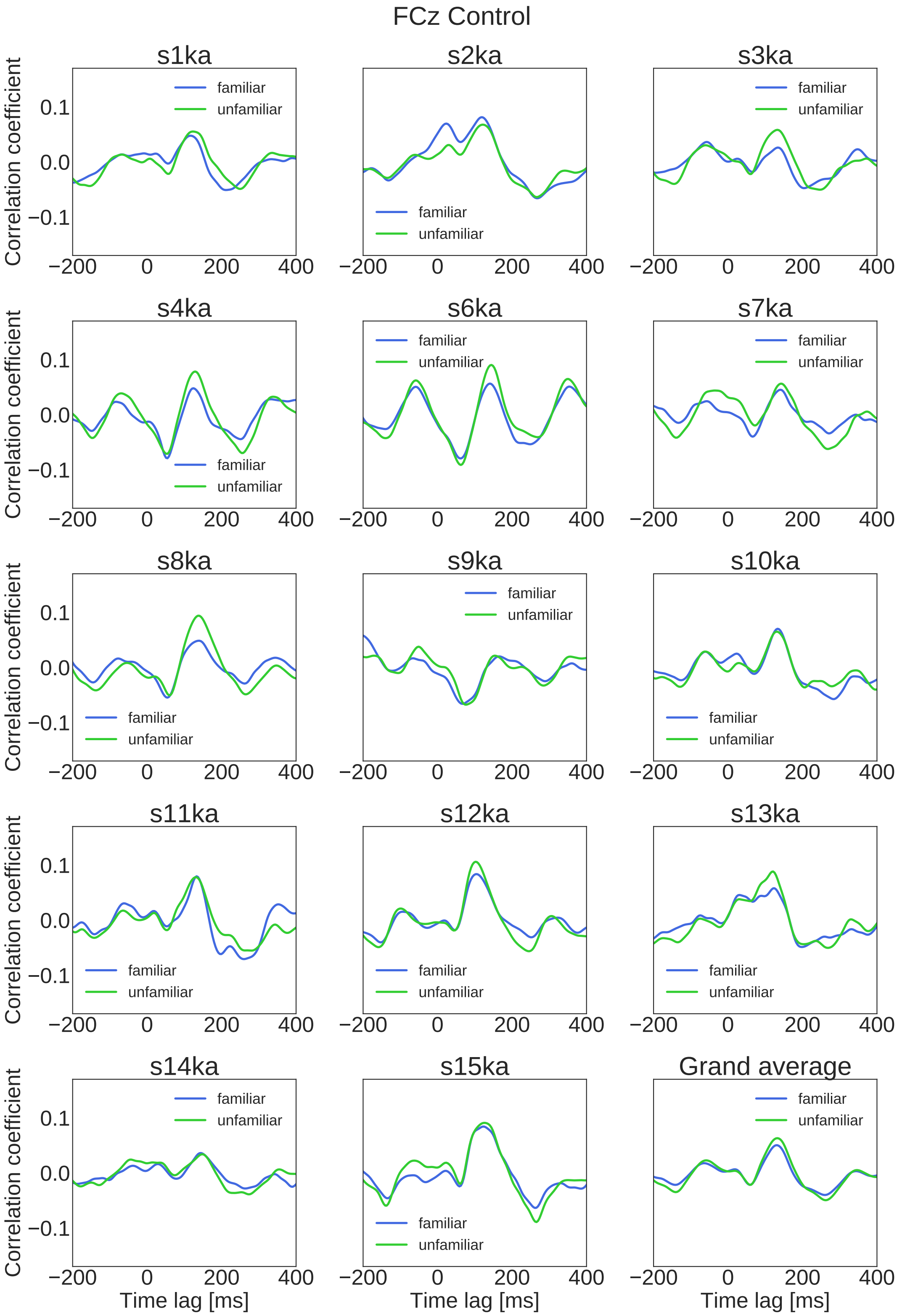

Figure S6. The averaged cross-correlation at FCz across trials in the control condition for each participant and the grand average across participants.

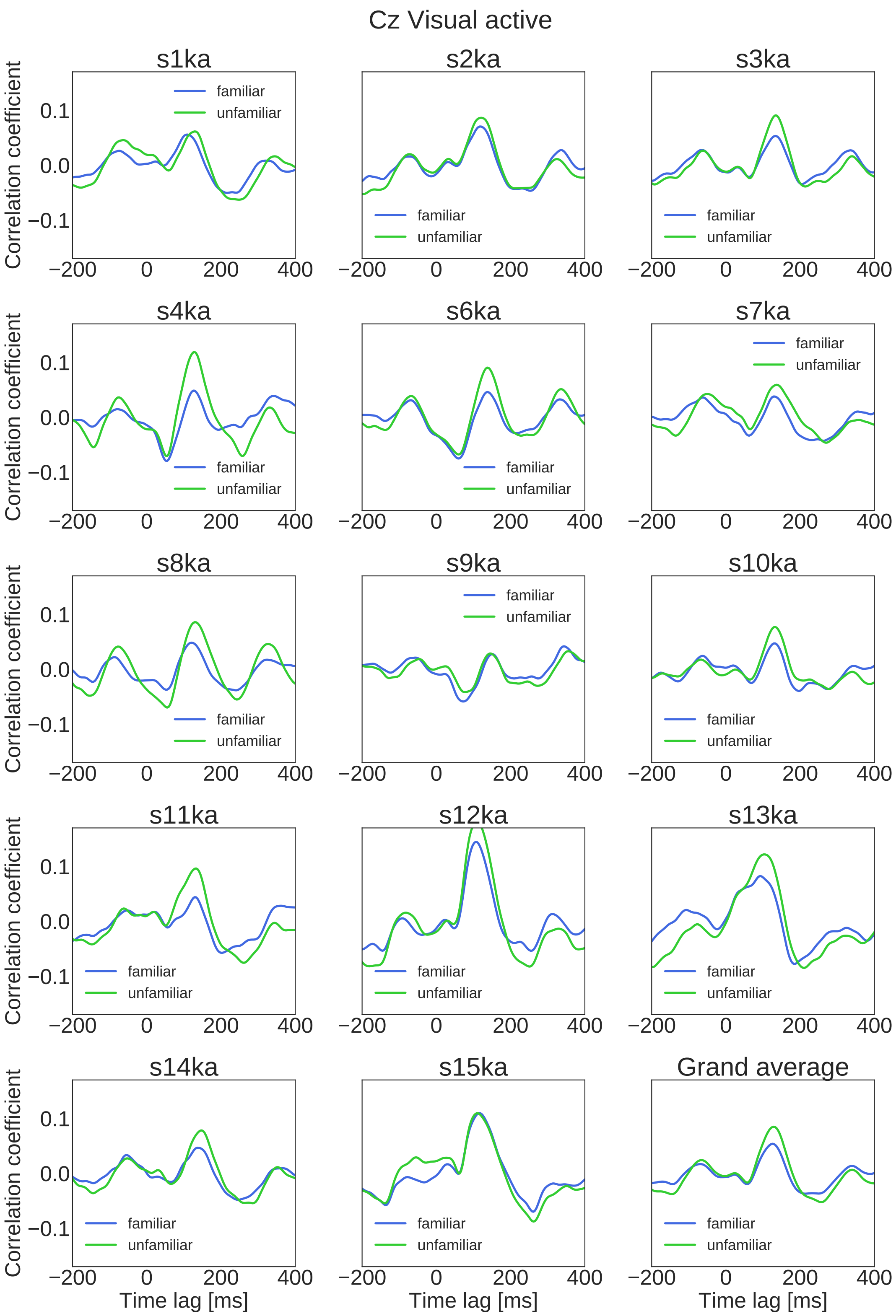

Figure S7. The averaged cross-correlation at Cz across trials in the visual-active condition for each participant and the grand average across participants.

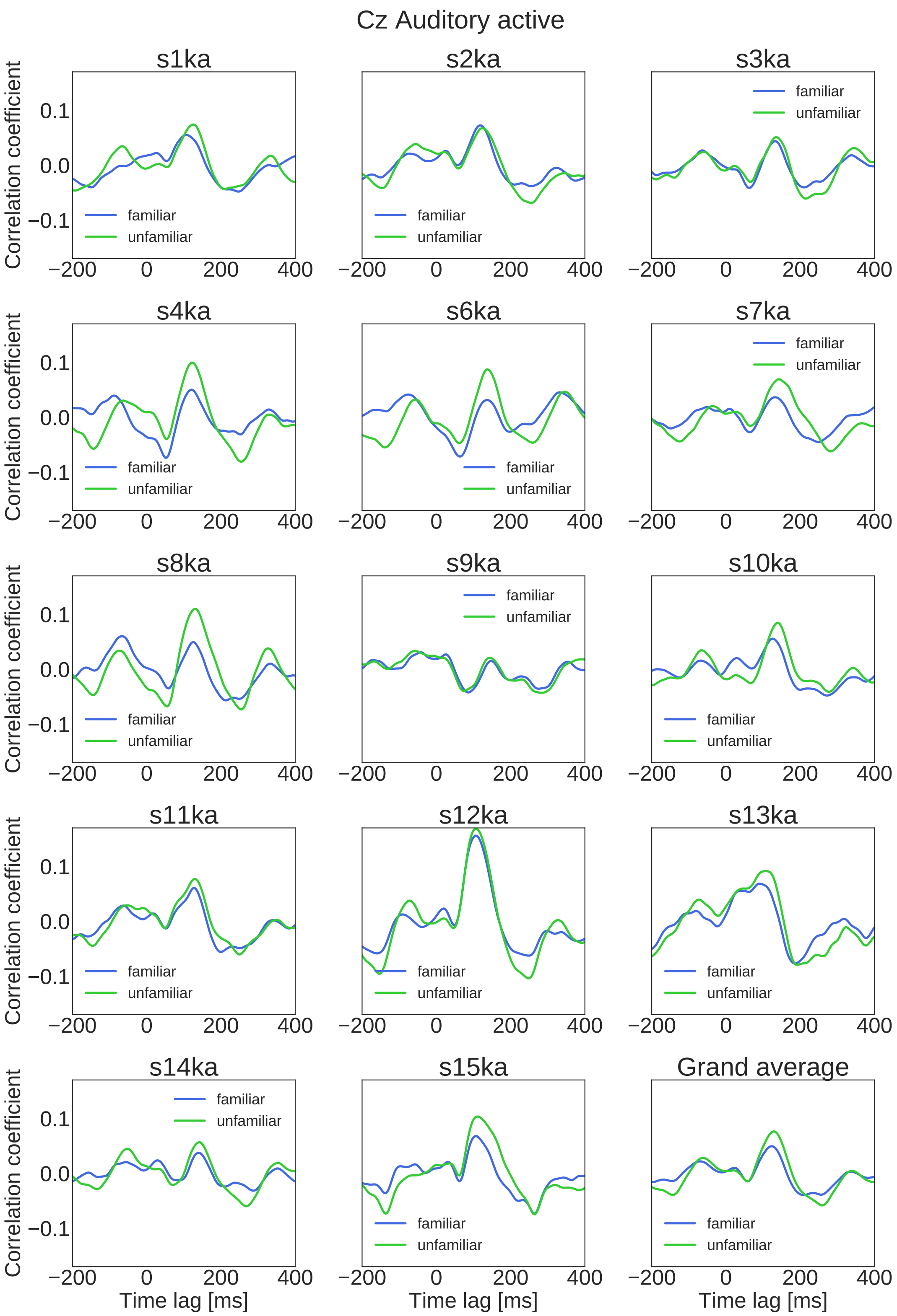

Figure S8. The averaged cross-correlation at Cz across trials in the auditory-active condition for each participant and the grand average across participants.

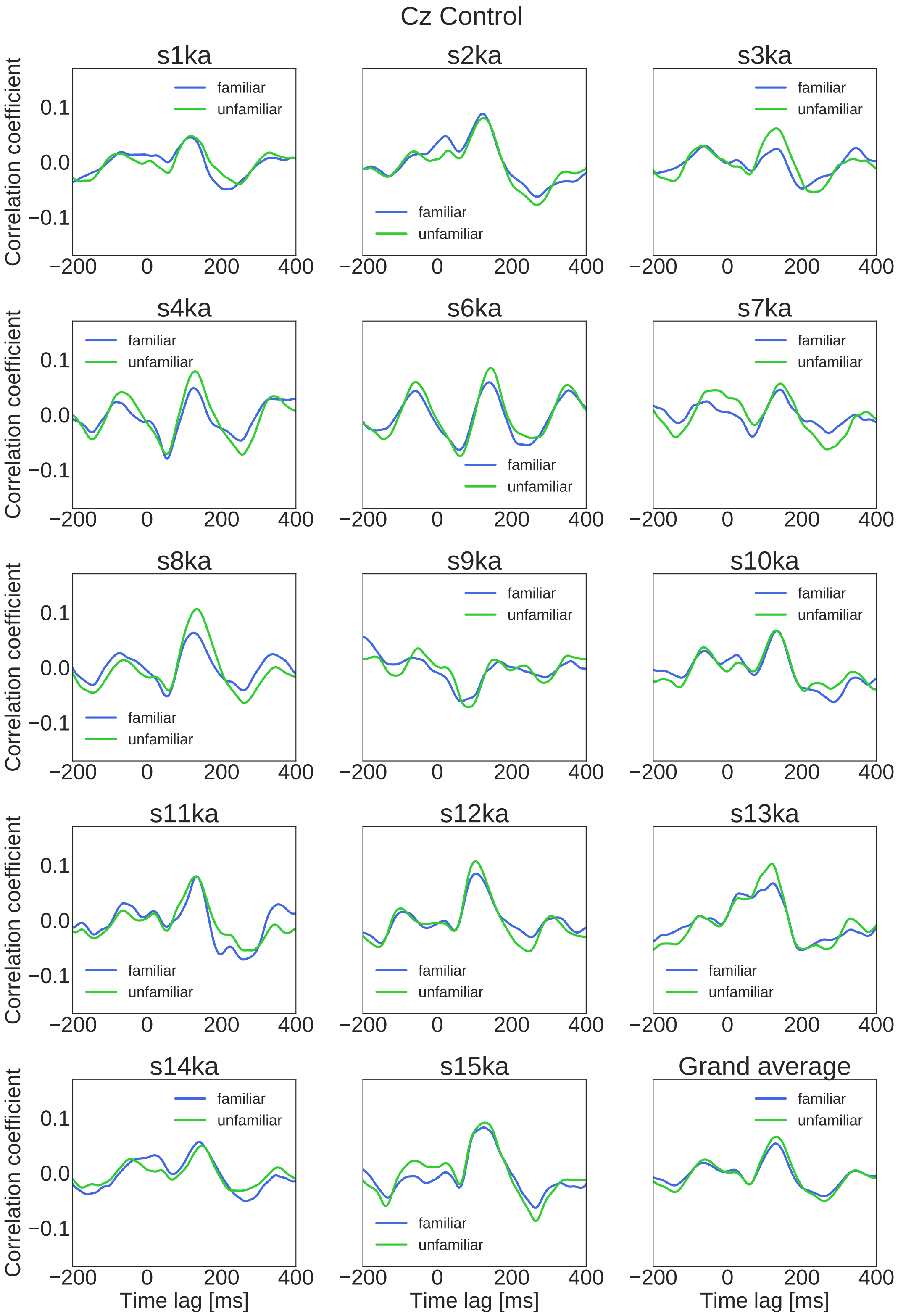

Figure S9. The averaged cross-correlation at Cz across trials in the control condition for each participant and the grand average across participants.

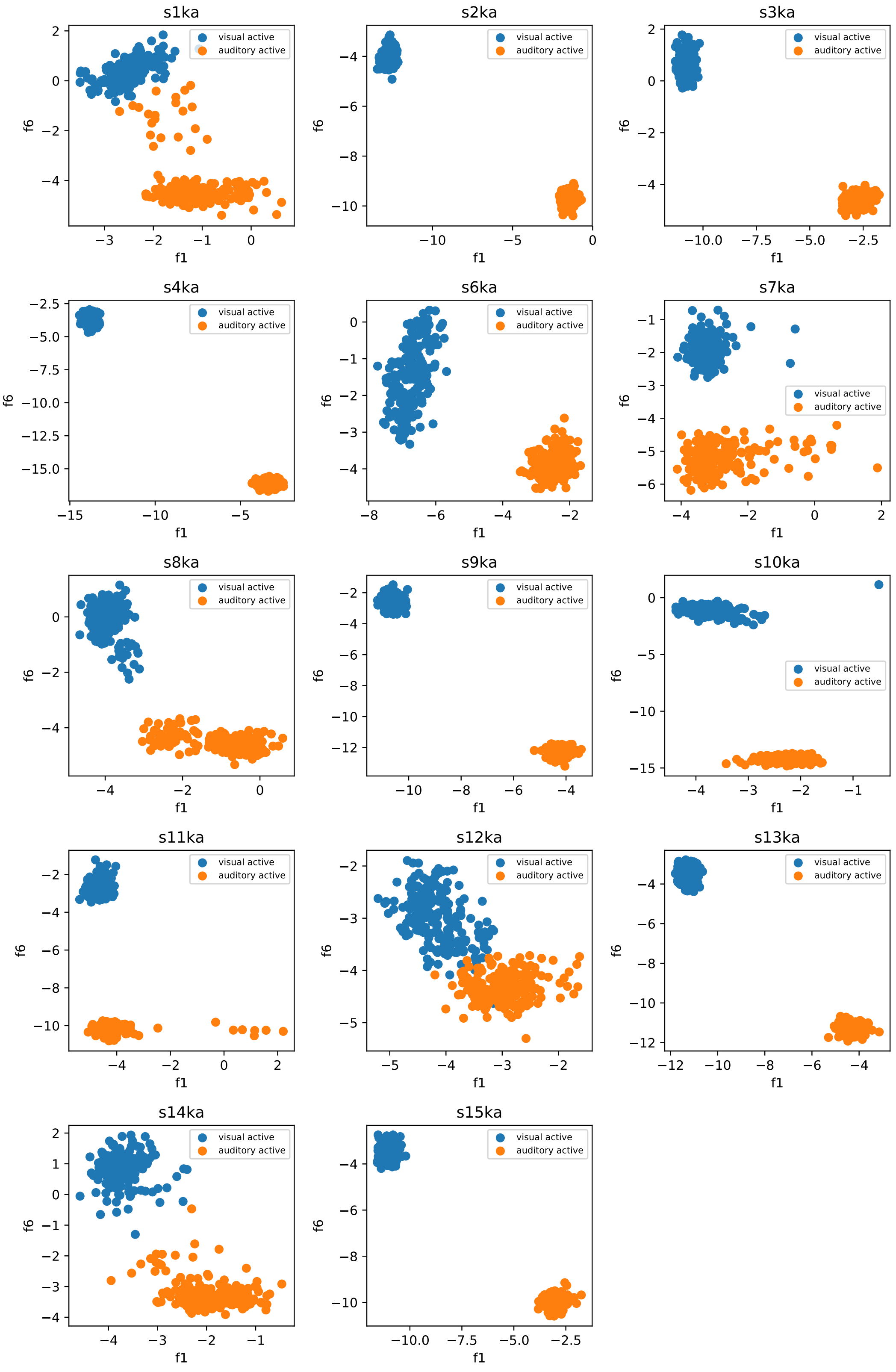

Figure S10. Scatter plots of the first and the sixth (last) components of feature vectors.

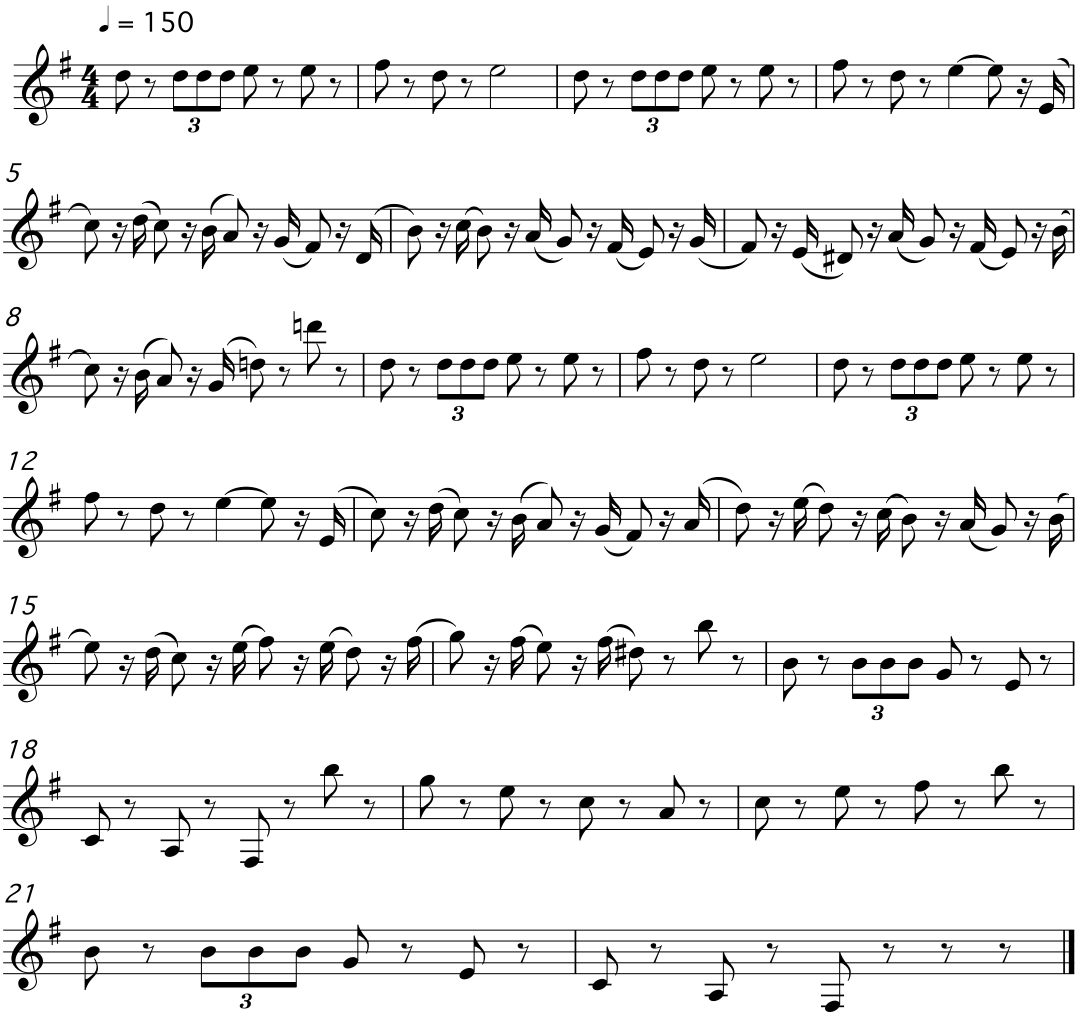

Figure S11. The synthesized musical notes of the March from Tchaikovsky's *The Nutcracker*.
